# Efficacy and safety of rituximab as second-line therapy in immune thrombocytopenic purpura based on ethnicity: A descriptive study among the Arabic population

**DOI:** 10.5339/qmj.2022.22

**Published:** 2022-07-07

**Authors:** Fateen Ata, Zohaib Yousaf, Fathima Z. Zahir, Anas Mohamed Babiker, Amer Ali Farooqi, Mousa Ahmad Al Hiyari, Adel Issam Al Bozom, Ahmed Hatim. Mohamed, Abdulqadir J. Nashwan, Mohamed A. Yassin

**Affiliations:** ^1^Department of Internal Medicine, Hamad General Hospital, Hamad Medical Corporation, Doha, Qatar E-mail: fata@hamad.qa; docfateenata@gmail.com; ^2^Department of Nursing for Education & Practice Development, Hamad Medical Corporation, Doha, Qatar; ^3^Department of Medical Oncology/Hematology, National Centre for Cancer Care and Research, Hamad Medical Corporation, Doha, Qatar

**Keywords:** Rituximab, Immune thrombocytopenic purpura, ITP, Ethnicity

## Abstract

Background: Rituximab is used as second-line therapy in patients with immune thrombocytopenic purpura (ITP) who do not respond to first-line management. The response rate for Rituximab is variable in different populations ranging from 30% to 90%. The adverse effects of rituximab in patients with ITP range from infusion site reactions to the reactivation of hepatitis B virus and progressive multifocal leukoencephalopathy and interpopulation variation.

Methods: We conducted a single-center, retrospective study in Qatar's National Center for Cancer Care & Research. The study included patients with chronic refractory ITP who received rituximab as second-line therapy. Descriptive and summary statistics were used to describe the sociodemographic parameters of the study cohort.

Results: Of the 41 patients with chronic ITP, 26 were Arabs, 12 were Asians, and 3 were of other ethnicities. Rituximab was associated with an overall response rate of 80.4%. Arabic patients had the highest clinical response (84.6%) among the ethnicities with the lowest adverse effects (11.5%). Asians had a response rate of 66.6%, and adverse effects were seen in 16.7% of the patients.

Conclusions: In chronic refractory ITP, rituximab appears to have a better clinical response in the Arabic population with minimal toxicity than in other ethnicities.

## Background

Immune thrombocytopenic purpura (ITP) is a benign hematological disorder that presents mainly with minor bleeding, petechial or purpuric rashes, and low platelet levels. However, it can get complicated with life-threatening bleeding, which is estimated to occur in 15% of patients. The overall prevalence of ITP is less studied given its rarity. Fogart et al. reported a prevalence of 5/100,000 children and 2/100,000 adults in a review of 2638 patients with ITP.^1^ Its pathophysiology has not been completely understood. However, most of the evidence linked it with the immune-mediated destruction of platelets (hence the name change from idiopathic to immune-mediated thrombocytopenic).^2^ The main mechanism of thrombocytopenic in ITP lies in the production of antiplatelet antibodies through B-cell-mediated immune response (mainly IgG and rarely IgM and IgA), causing a breakdown of the platelets in the spleen and liver.^3^ These antibodies bind to the surface of the platelets to GPIIbIIIA and GPIb-IX-V molecules. However, in up to 40% of cases, no antibodies are isolated, which may be due to a T-cell-mediated mechanism.^3^

Rituximab is an anti-CD20-antibody that was initially approved for the treatment of B-cell lymphoma. Its mechanism of action lies in the destruction of B cells. Apart from lymphomas, rituximab is currently used in various clinical conditions, including autoimmune disorders such as rheumatoid arthritis, autoimmune skin conditions, Sjogren syndrome, and vasculitis.^4^ Additionally, rituximab has proven efficacy as a second-line treatment for acute ITP in at least five studies.^5^ However, data regarding long-term sustained response is limited.^6^ The effectiveness of rituximab in ITP is based on the finding that it depletes antiplatelet antibodies in vivo, thus countering the main pathophysiological mechanism of thrombocytopenic in ITP.^7^ It is given as a weekly injection (375 mg/m^2^), and most of the patients require at least four doses. Response to rituximab in the Western population is approximately 62%.^8^ The efficacy and safety of rituximab based on ethnicity have been examined before, such as in the Japanese population. In a study of 26 patients with ITP, response (platelet count > 50 × 10^9^/L) was achieved in 30.8%.^9^ However, the response to rituximab of patients with ITP and Middle Eastern ethnicity has not been adequately assessed before and is limited to a case series of 12 patients.^10^ In this study, we conducted a retrospective examination on 41 patients diagnosed with ITP and were treated with rituximab to ascertain the drug's efficacy and safety based on their ethnicity.

## Research design and methods

### Study design

We conducted a retrospective cross-sectional data analysis of patients with chronic ITP (primary or secondary) who were admitted between January 2015 and December 2020. These patients were refractory to standard first-line management and received rituximab as the second-line management for ITP. This study was conducted at the Weill Cornell Medicine affiliated- National Center for Cancer Care & Research, Hamad Medical Corporation, Doha, Qatar,

### Inclusion and exclusion Criteria

All adult patients (aged >18 years) with chronic ITP either admitted to or visited the daycare unit who received rituximab as a second-line therapy between 2015 and 2020 were included in the study.

The patient cohort included adults who had a prior diagnosis of chronic ITP (>12 months) made by a hematologist. These patients either had a failed response to the first-line treatment (corticosteroids or intravenous immunoglobulin) or did not receive them because of contraindications. All patients received rituximab at a dose of 375mg/m^2^ as a weekly infusion for 4 weeks. First, patients who had other concomitant malignancies or had other indications of rituximab were excluded from the study. Second, patients who received concomitant medication that could increase platelets, including immunoglobulins and platelet-stimulating agents, were also excluded. Third, patients who had concurrent infections which could potentially lead to reactionary thrombocytosis were also excluded from the analysis. Finally, three patients who were initially diagnosed with ITP were found to have unexplained splenomegaly on ultrasonography of the abdomen; hence, they were removed from the cohort.

Data of the patients with chronic ITP admitted between 2015 and 2020 were abstracted from the electronic records of Hamad Medical Corporation patient data repository (Cerner). Data collected included demographics such as age, sex, ethnicity, body mass index (BMI), and relevant laboratory investigations on admission. Data related to ITP status included platelet level, hemoglobin level, and mean platelet volume on admission. The dose and duration of rituximab were obtained from the medication charts and discharge summaries of the included patients. Data collected for response assessment included relevant clinical observations such as major or minor bleeding; laboratory investigations such as platelet level, hemoglobin level, and mean platelet volume after treatment; adverse effects of rituximab; and mortality. The response was calculated 4 weeks after the last infusion of rituximab. A period of 4 weeks was chosen based on the median response rate to rituximab in previous studies of patients with ITP.^11^ The response criteria were described based on the platelet levels used by Stasi et al. in their study^12^: complete response (CR) (>100 × 10^9^/L), partial response (PR) (51–100 × 10^9^/L), minor response (MR) (31–50 × 10^9^/L), and no response (1–30 × 10^9^/L), in accordance with the previously used criteria to keep the results comparable with those of previous studies.

### Statistical analyses

Descriptive and summary statistics were used to describe the sociodemographic parameters of the study cohort, with continuous variables in mean with standard deviation or median along with interquartile range as appropriate. The Shapiro–Wilk test was used to check the normality of data distribution. Categorical variables were reported as numbers with percentages. All data were analyzed using Jamovi version 1.2 (created in 2020, Sydney, Australia).^13^

### Ethical considerations

This study was conducted in compliance with the Declaration of Helsinki, Good Clinical Practice, and other relevant regulatory requirements. The institutional review board in Medical Research Center (MRC) Qatar reviewed the ethical, medical, and scientific aspects and approved the study (MRC-01-20-577).

## Results

### Demographics

Table 1 summarizes the demographics of the patient population. A total of 41 patients were included in the analysis after the application of the inclusion and exclusion criteria; 24 (58.1%) were male, whereas 17 (41.5 %) were female. Qataris accounted for the highest number of patients (N = 10, 24.4%), followed by Indians (N = 6, 14.6%), Egyptians (N = 4, 9.8%), Jordanians (N = 3, 7.3%), Bangladeshis (N = 3, 7.3%), Sudanese (N = 2, 4.9%), Syrians (N = 2, 4.9%), Pakistani (N = 2, 4.9%), Canadian (N = 1, 2.4%), British (N = 1, 2.4%), Iranian (N = 1, 2.4%), Moroccan (N = 1, 2.4%), Palestinian (N = 1, 2.4%), Saudi Arabian (N = 1, 2.4%), Filipino (N = 1, 2.4%), Somalian (N = 1, 2.4%), and Romanian (N = 1, 2.4%). Ethnicities were divided into Arabics (N = 26, 63.4%), Asians (N = 12, 29.3%), and others (N = 3, 7.3%). The median platelet level upon admission was 15 (8–23) × 10^9^/L, and 15 (36.5%) patients had an incidental finding of ITP without any evidence of bleeding. Moreover, 13 (31.7%) patients had cutaneous bleeding at presentation, and 9 (21.9%) had mucosal bleeding episodes upon presentation. In addition, 4 (9.7%) patients had a life-threatening bleeding episode as a presentation of ITP (two with vaginal bleeding and one with subdural hemorrhage). Antinuclear antibody (ANA) was positive in 11 (28.2%) patients on admission. All patients received rituximab based on the standard dose of 375 mg/m^2^ weekly infusion. The mean dose was 678 ± 77 mg, whereas the median duration of rituximab therapy was 4 (4–6) weeks.

### Efficacy analysis

A total of 33 (80.4%) patients had a good response to rituximab therapy (platelet level above 51 ×  10^9^/L post-treatment). Among these, 9 (22%) had platelet levels above 51–100 × 10^9^/L (PR), whereas 24 (58.5%) had platelet levels above 100 × 10^9^/L (CR). On the contrary, 8 (19.1%) patients did not respond well to rituximab therapy (including poor and no response). The median platelet level after rituximab therapy was 138 (IQR 71–234) × 10^9^/L, which increased from the baseline median of 15 (IQR 8–23) × 10^9^/L. Table 2 represents the response to rituximab in patients with ITP based on ethnicity. Moreover, 22 of 26 (84.6%) Arabic patients had a good clinical response to rituximab (platelet level above 51 ×  10^9^/L post-treatment), and 17 (65.4%) Arabic patients had CR with platelets > 100 × 10^9^/L post-treatment. Among the 12 Asian patients, 8 (66.6%) had a good clinical response, including 4 (33.3%) patients with CR (platelets >100 × 10^9^/L) post-treatment. Although the number of patients with ethnicities other than Arabic and Asian was limited (N = 3), a remarkable clinical response (platelets > 100 × 10^9^/L post-treatment) was seen in all patients.

### Safety analysis

Rituximab therapy was safe in our patient population with no adverse reaction in 35 (85.4%) patients. Others reported side effects that were minor and did not require admission, extended hospital stay, or any intervention. These included shortness of breath (N = 2, 4.9%), mild allergic reaction (N = 1, 2.4%), throat irritation (N = 1, 2.4%), generalized pain (N = 1, 2.4%), and headache and rash (N = 1, 2.4%). Arabic patients had the lowest occurrence of adverse effects with rituximab (N = 3, 11.5%), followed by Asians (N = 2. 16.7%) and other ethnicities (N = 1, 33.3%) [Table 3]. In our patient cohort, 15 (37.5%) had evidence of hepatitis B virus infection (positive core antibody, surface antibody, polymerase chain reaction [PCR], and antigen levels where available). However, none of the patients had an active disease during the rituximab treatment or antiviral therapies. We compared the antibody and antigen results and PCR values where available, before and after rituximab therapy. None of the patients had evidence of reinfection.

## Discussion

This study represents the first comprehensive evaluation of the efficacy and safety of rituximab in Qatar's population. Our patient cohort comprises 41 patients with chronic ITP refractory to first-line treatment (steroids or immunoglobulins). We found a good overall clinical response to rituximab (80.4%). These patients were not on any other immunosuppressive medication during the period of treatment with rituximab; hence, the response can be associated with the effects of rituximab. Prospective studies have shown the efficacy of rituximab at approximately 60% in patients with chronic ITP.^14^ The most extensive data regarding the efficacy of rituximab in refractory ITP comes from a systematic review conducted in 2017, in which patients with ITP demonstrated a response rate of 60%. However, many adverse effects and deaths were reported, and no definite conclusions could be made on risks relative to the benefits of rituximab in chronic ITP in a diverse population.^8^ In situations like this, ethnicity-based evaluation of the efficacy and safety of drugs can be a valuable tool for precision medicine until more extensive clinical trials are conducted with a heterogeneous population. Differences in drug effectiveness and safety based on ethnicity are well-established concepts.^15^ Although delineating the factors behind such variations is complex, environmental factors, such as smoking, alcohol consumption, and weather, may play a significant role. Other important factors may include cultural variations and psychosocial practices, which can influence drug response in the human body.^15^ Genetic variations also play an essential role in how the disease affects people from a specific background. These differences also translate to a varied drug response targeting such diseases.^16^ This is similarly true for ITP, which shows diversity in clinical presentations based on ethnicity. In 2020, Kim et al. studied the variation in the prevalence of ITP in African children due to biological differences. The authors concluded that African children were less prone to ITP. However, those who developed ITP tended to have a more chronic course than other ethnicities.^17^ Considering the above known and other unknown factors, differences in drug effectiveness and safety based on ethnicity are a growing interest in medical research, as the world goes toward personalized medicine.^18^

Growing evidence presents the genetic influence on the responsiveness of rituximab in various diseases. This can be partly associated with the mechanism of action of rituximab. Upon binding to B cells, rituximab initiates complement-dependant (CDC) and antibody-dependant cytotoxicity (ADDC). In the CDC, the complement cascade is activated following the binding of C1q to the drug molecule. In ADDC, phagocytosis of B cells is triggered by immune effector cells that identify and target the Fc portion of the bound anti-CD-20 antibody. Polymorphism in the alleles responsible for the formation of binding receptors is one reason for the varied response to rituximab in diseases based on ethnic differences.^19^

Miyakawa et al. investigated the efficacy and safety of rituximab in Japanese patients with chronic refractory ITP. Rituximab was given at a dose of 375 mg/m^2^, as once-weekly infusion for 4 consecutive weeks. They reported a response rate of 30.8% (platelet level ≥ 50 × 10^9^/L), calculated at week 24 from the start of rituximab therapy.^9^ The Japanese study included a sample of 26 patients. Although small, their sample size was adequate for an ≥ 80% power to assess the response rate to rituximab. Another study conducted in Italy described an overall response rate (CR + PR) of 40% in a cohort of 25 patients with chronic ITP.^12^ The sample size in both studies was comparable with different results. Hence, we can deduce that the difference in response rates may not be due to the sample size but different ethnic origins of the patient population. Cooper et al. reported a response rate of 54% (platelet level ≥  50 × 10^9^/L) to rituximab in patients with refractory ITP. They compared two populations, the USA and Italy, and reported combined results, as they found similar response rates in both.^20^ In another study on 12 German patients with ITP, Giagounidis et al. reported a response rate of 58% to rituximab (CR and PR combined).^21^ Notably, ethnic variation may have existed in their patient population, as ethnicity was not specified in these studies.(20, 21)

A small prospective study on 12 Kuwaiti patients with ITP (excluding the two with Evans syndrome) reported a response rate of 91.6% (CR in 10 and PR in 2) to rituximab in refractory ITP.^10^ The results concerning the efficacy are comparable to our study's Arabic population (84.6% CR and PR combined), with some differences associated with the difference in the study type and sample size. The adverse effect rate in Kuwaiti patients was 35.7%, and adverse reactions reported were infusion-related toxicity and sepsis. Compared with the results reported by Alasfoor et al., the Arabic population in our study had better safety outcomes (11.5% adverse reactions in our study vs. 35.7% in Alasfoor et al.).^10^ Keeping with the Kuwaiti study results, the response to rituximab did not appear to be dependent on the duration of ITP. From the diagnosis to rituximab treatment, our patients’ duration ranged from 1 year to 30 years. No significant difference was found in response rates based on the disease duration.

When comparing the studies on the Arabic population with other ethnicities, findings suggest that ethnicity can significantly affect the response rate to rituximab in refractory ITP. However, more extensive prospective studies are necessary to validate these results.

The long-term efficacy and safety of rituximab in refractory ITP is a relatively underexplored area. A recent study published in 2019 reported 5-year efficacy and safety data for rituximab in 248 patients with ITP. The long-term sustained response rate was 29.4%. The study also reported retreatment in patients who initially received rituximab but relapsed. Interestingly, the response rate to rituximab is much higher (92%). The study reported adverse effects of various grades, with a cumulative rate of 54.4%. However, severe reactions (grades 4 and 5) were seen in 9.7% of the patients. Mortality was reported in 10.1% of patients, with a mortality rate of 2.3/100 patient-years. Infections led to 5 deaths; however, the authors associated three of them with rituximab.^22^

Rituximab infusions have been associated with the reactivation of hepatitis B infection in previous studies.^14^ In patients who received rituximab for ITP, there is limited evidence about the reactivation of hepatitis B or other viral infections. In our patient cohort, 37.5% of the patients had a history of hepatitis B infection. None of the patients had a rise in the PCR or change in the antigen status post-treatment. Hence, rituximab therapy for patients with ITP may not lead to the reactivation of viral illnesses.

Table 4 shows a comparison of the response rates of patients with chronic ITP from various ethnic origins to rituximab. Therefore, there may be a general trend of a better response rate and a better safety profile in Arabs.

The response to drugs based on ethnicity is an emerging and crucial aspect of cost-effectiveness and individualized pharmaceutical management, especially when the drug in question is expensive and the disease is chronic. The reason behind such differences is multifactorial, mainly comprising of genetic differences leading to variations in disease manifestations and drug effects. Additional factors can be related to environmental, psychosocial, and cultural aspects.^15^ The earliest strong evidence regarding varying response rates based on ethnic diversity came from a study that concluded that the Caucasian population had a better response to propranolol in controlling blood pressure than the African population.^23^ The most notable ethnicity-based difference was noted with angiotensin-converting enzyme inhibitors.^24^ A similar approach is being used in detecting responsiveness based on ethnicity with other diseases such as asthma, schizophrenia, depression, among others.(25-27)

This study has some limitations inherent to retrospective chart reviews. First, some of the confounding factors that could affect platelet rise could not be assessed, such as contamination with self-medication and unreported mild infections. Similarly, unreported and self-limiting mild side effects, which were not reported, could not be analyzed for their presence or absence. The principal strength of this study lies in its novelty for analyzing the efficacy and safety of rituximab in ITP for the Arabic population in a relatively larger patient cohort.

## Conclusion

Rituximab has varying efficacies in chronic ITP in patients of different ethnicities, ranging from 30.8% to 91.6 %. Arabic patients with ITP appear to have a better response rate to rituximab with lower adverse effects than other ethnicities. More extensive studies on the efficacy and safety of rituximab in Arabic patients with ITP can validate our results, which will consequently enhance the guidelines regarding the use of rituximab in ITP.

## Declarations

### Ethics approval and consent to participate

Ethical approval for this study was obtained from Medical Research Center (MRC) Qatar (MRC-04-21-100). Consent was not required, as this was a retrospective chart review of patients with no sharing of any identifiable data.

### Consent for publication

This was not required, as this was a retrospective chart review of patients with no sharing of any identifiable data.

### Availability of data and materials

The datasets used and/or analyzed during the current study are available from the corresponding author on reasonable request

### Competing interests

The authors declare that they have no competing interests.

## Figures and Tables

**Table 1:** Demographics of patients with immune thrombocytopenic purpura (ITP) who received tituximab treatment.

Characteristics	Results (N = 41)

Age at the time of rituximab administration (years) (mean ± SD)	38.2 ± 14.4

Age when diagnosed with ITP (years) (mean ± SD)	34.6 ± 14.8

Weight (kg) (mean ± SD)	76.7 ± 15.3

BMI (kg/m^2^) (mean ± SD)	28.6 ± 5.36

Ethnicity (N, %) Arabs Asians Others	26 (63.4%) 12 (29.3%) 3 (7.3%)

Sex (N, %) Male Female	24 (58.5%) 17 (41.5%)

PLT on admission (x 10^9^/L) (median with IQR)	15 (8–23)

Hgb on admission (g/dL) (mean ± SD)	12.6 ± 2

WBC on admission (x 10^9^/L) (median with IQR)	6.4 (4.5–9)

MPV on admission (fL) (median with IQR)	10.9 (9.35–11.7)

ANA (N = 39) Positive Negative	11 (28.2%) 28 (71.8%)

Hepatitis B (N = 40)	15 (37.5%)

Rituximab dose (mg) (mean ± SD)	678 ± 77

Rituximab treatment duration (weeks) (median with IQR)	4 (4–6)

Adverse events (N = 41) None reported. Mild allergic reaction Throat irritation Shortness of breath Generalized pain. Headache and rash	35 (85.4%) 1 (2.4%) 1 (2.4%) 2 (4.9%) 1 (2.4%) 1 (2.4%)

PLT response post-treatment (N, %) 1–30 x 10^9^/L 31–50 x 10^9^/L 51–100 x 10^9^/L >100 x 10^9^/L	6 (14.6%) 2 (4.9%) 9 (22%) 24 (58.5%)

PLTs post-treatment (x 10^9^/L) (median with IQR)	138 (71–234)

MPV post-treatment (fL) (mean ± SD)	10.7 ± 1.87

Hgb post-treatment (g/dL) (mean ± SD)	12.8 ± 2.22

WBC post-treatment (x 10^9^/L) (median with IQR)	7.4 (5.97–9.1)


(Hgb, hemoglobin; PLT, platelets; WBC, white blood cell; SD, standard deviation; IQR, interquartile range; MPV, pean platelet volume; HIV, human immunodeficiency virus; BMI, body mass index; ANA, antinuclear antibody)

**Table 2:** Response rate to rituximab based on ethnicity. Presented as numbers with percentages and median with interquartile ranges (IQR), where applicable.

Efficacy of rituximab	Total (N = 41)	Arabic (N = 26)	Asian (N = 12)	Others (N = 3)

Platelets on admission (median with IQR)	15 (8–23)	15.5 (11–32.8)	12 (7.75–23)	5

Platelet response 1–30	6 (14.6%)	4 (15.4%)	2 (16.7%)	0

Platelet response 31–50	2 (4.8%)	0	2 (16.7%)	0

Platelet response 51–100	9 (21.9%)	5 (19.2%)	4 (33.3%)	0

Platelet response >100	24 (58.5%)	17 (65.4%)	4 (33.3%)	3 (100%)

Platelets post-treatment (median with IQR)	138 (71–234)	163 (71.3–304)	89 (32–139)	200


**Table 3:** Safety profile of rituximab in the study cohort. Presented as numbers with percentages.

Safety of rituximab	Arab (N = 26)	Asian (N = 12)	Others (N = 3)

Total adverse events	3 (11.5%)	2 (16.7%)	1 (33.3%)

Mild allergic reaction	1 (3.8%)	2 (16.7%)	0

Shortness of breath	2 (7.7%)	0	0

Throat irritation	0	0	1 (33.3%)

Generalized pain	0	1 (8.3%)	0


**Table 4:** A comparison of responsiveness to rituximab of patients with chronic ITP of different ethnicities, according to previously published studies.

Study	N	Author, Year	CR	PR

Italian (12)	25	Stasi et al., 2016	5 (20%)	5 (20%)

Japanese (9)	26	Miyakawa et al., 2015	4 (15.4 %)	8 (30.8 %)

French (22)	248	Deshayes et al., 2019	80 (32.3%)	NA

Italian and USA (20)	57	Cooper et al., 2004	18 (31.5%)	13 (22.8%)

Kuwaiti (10)	12	Alasfoor et al., 2009	10 (83.3%)	2 (16.6%)

Our study (Arabic)	26		17 (65.4%)	5 (19.20%)

Our study (Asian)	12		4 (33.3%)	4 (33.3%)


(N, number of patients in the study; CR, complete response; PR, partial response) (Note: The CR in all studies was platelet level >100, and PR in all studies was platelet level >50, except Cooper et al., where CR was platelet level >150, and PR was platelet level between 50 and 150.)
